# Bacteraemia and antibiotic sensitivity in a tertiary neonatal intensive care unit

**DOI:** 10.4102/sajid.v36i1.195

**Published:** 2021-01-05

**Authors:** Hamida van Staaden, Candice Hendricks, Kevin Spicer

**Affiliations:** 1 Department of Paediatrics and Child Health, Nelson R. Mandela School of Medicine, Faculty of Health Sciences, University of KwaZulu-Natal, Durban, South Africa; 2 Department of Paediatrics, Pietermaritzburg Metropolitan Hospitals Complex, KwaZulu-Natal Department of Health, Pietermaritzburg, South Africa

**Keywords:** neonatal sepsis, positive blood cultures, antimicrobial resistance, antibiotic resistance, culture collection processes

## Abstract

**Background:**

Neonatal sepsis is an important cause of mortality and morbidity in neonatal intensive care populations worldwide. Data on rates of bacteraemia and antibiotic resistance patterns are limited, particularly in the developing world.

**Methods:**

We retrospectively reviewed positive blood cultures obtained in the neonatal intensive care unit between 01 January 2015 and 31 December 2015. All neonates, either born at the tertiary hospital or transferred from referral units, regardless of diagnosis, who had a positive blood culture were included.

**Results:**

There were 702 admissions during the study period and 437 positive cultures. Male patients made up 55.1% (65/118), and the gender was unknown for 11.0% (13/118). Late onset sepsis accounted for 85.7% (102/119) and early onset sepsis, 14.3% (17/119). Of the 119 organisms cultured, 76 (63.8%) were Gram-negative, 35 (29.4%) were Gram-positive and 8 (6.7%) were *Candida* species. *Klebsiella* was the most common genus at 42% (50/119). Of the clinically relevant organisms recovered, 37.0% (44/119) were susceptible to the empiric first-line regimen of penicillin and gentamycin. Furthermore, 69.7% (53/76) of the Gram-negative organisms produced extended-spectrum beta-lactamases.

**Conclusion:**

The majority of organisms cultured were considered contaminants and were not clinically relevant. Improvements in culture collection processes are needed. The majority of organisms considered clinically relevant were resistant to the first-line antibiotic regimen. To improve the likelihood of clinical success, empiric antibiotic regimens should be based on local data, if possible.

## Introduction

Neonatal sepsis is an important cause of mortality and morbidity in neonatal intensive care unit (NICU) populations worldwide. The World Health Organization reports that more than 4 million babies die in the first 28 days of life each year and that infections and sepsis, respectively, account for 36% and 6% of these neonatal deaths.^1^ Neonates, and particularly preterm newborns, are at higher risk of sepsis.^2,3^ Amongst the factors implicated are impaired cytokine production, reduced expression of adhesion molecules in neutrophils and reduced cytotoxic T-cell activity.^4^ Coupled with invasive interventions often carried out in the NICU, this leaves neonates at a considerably high risk for sepsis.^4,5,6^ Individual patient morbidity is not the only factor to consider. Neonates with suspected or proven sepsis require longer hospital stays, resulting in added cost and decreased turnover of beds.^7,8^ This is particularly problematic in the developing world with overcrowded nurseries.^9^ These pressures lead to liberal use of antibiotics and often rapid escalation to higher classes of drugs.^9^ Subsequently, organ dysfunction, ototoxicity and, more importantly, the development of antibiotic resistance become major factors to consider.^10,11^

Diagnosis and early treatment of neonatal sepsis remain challenging, as the signs and symptoms of neonatal sepsis are often non-specific.^9^ Presentation varies and can include temperature instability, feeding difficulty, respiratory distress, lethargy and seizures.^9,12^ Prompt and efficient diagnosis and appropriate treatment are vital to prevent ongoing morbidity and mortality. Blood cultures are frequently performed in the NICU either on arrival to the unit or when there is clinical deterioration.^13^ Blood cultures, despite lacking 100% sensitivity, remain the gold standard for confirmation of a diagnosis of septicaemia.^4,14^ Additionally, identification of the causative organism allows for antibiotic susceptibility testing and provides a basis for targeted, and more effective, antibiotic therapy. Organisms that are more commonly implicated in neonatal sepsis differ between developing and developed countries, and the former has been shown to have more healthcare-associated infections.^15^ Overall, Gram-negative organisms are more common and include *Klebsiella* species, *Escherichia coli, Pseudomonas* species and *Salmonella* species.^2,7,15,16^ Gram-positive organisms most commonly isolated include *Staphylococcus aureus*, coagulase-negative staphylococci (CONS) and *Streptococcus pneumoniae*.^2,15^

A common practice is to start empiric antibiotics until sepsis is proven or excluded.^13^ This leads to widespread use of antibiotics as blood cultures often take days to identify an offending organism. Knowledge of the most common aetiological agents causing neonatal infection in a particular NICU is thus essential in developing strategies to prevent and treat serious neonatal infections in that unit. More effective prevention and treatment strategies will improve progress towards Millennium Development Goal 4, which targets mortality rates in children < 5 years of age.^17^ Empirical treatment protocols are often formulated from data gathered in developed countries. The NICU at the tertiary hospital uses soluble penicillin and gentamycin as first-line empirical treatment for infants with suspected infection or sepsis. With increasing resistance to penicillin and gentamycin, the need for information on local antibiotic susceptibility patterns is crucial.^3,5^

The aim of this study was to determine the prevalence of positive blood cultures and describe the identified organisms and associated antibiotic susceptibility patterns in the NICU of a tertiary hospital. This will contribute towards the formulation of local empirical antibiotic regimens and will illustrate an approach that can be taken by other facilities.

## Methods

This was a retrospective descriptive study, reviewing data from a tertiary NICU in KwaZulu-Natal, South Africa, for a 1-year period, from 01 January 2015 through 31 December 2015.

The NICU is a 26-bed tertiary unit that serves the neonatal population of Pietermaritzburg and referrals from Area 2 (comprising five districts with 20 hospitals) within KwaZulu-Natal, South Africa. The NICU consists of 6 beds for ventilation and 20 beds for neonatal high care. Care is provided to both medical and surgical patients.

All neonates, either born at the hospital or transferred from referral units, regardless of diagnosis, who had a positive blood culture were included in the study. Positive blood cultures were extracted from the National Health Laboratory Service (NHLS) computerised database. Information on patient demographics, dates when blood cultures were taken, organisms identified and antibiotic susceptibilities was collected. Data were entered into an Excel spreadsheet.

Patients were assessed for sepsis, and aseptic techniques were used to obtain blood cultures on admission as well as during periods of clinical deterioration. These episodes included, but were not limited to, periods of temperature instability, respiratory distress and need for ventilation and seizures. Blood volumes of 1 mL–2 mL were obtained from one site in each patient (unless a central line was present when two samples were considered at the discretion of the treating physician) and inoculated into one paediatric blood culture bottle (BACTEC Peds Plus Culture bottle). Samples were transported to the NHLS Microbiology Laboratory for incubation and were subsequently processed for susceptibility testing. The BACTEC instrument incubates and monitors the bottle for growth by detecting change in pH in the indicator at the bottom of the bottle. Changes in pH signal growth, and the bottle is flagged as positive. The positive blood culture bottle is then removed from the instrument, a Gram stain is made and the bottle contents are subcultured onto agar plates. The bottles remain in the instrument for 5 days after which they are removed if no growth occurs. The VITEK system (Vitek instrument for sensitivity testing, which uses the broth microdilution method) was used for organism identification and for ascertaining antibiotic susceptibility of recovered organisms.

Initiation of and changes in antibiotics were made by the attending paediatrician or neonatologist in the NICU. The antibiotic policy in the unit for infants with suspected infection or sepsis is as follows: first-line empirical therapy is soluble penicillin and gentamycin; second-line antibiotics include Tazocin (piperacillin-tazobactam) and amikacin, which have broader spectrum cover against most Gram-positive and Gram-negative aerobic and anaerobic bacteria, and third-line treatment is meropenem, which has the broadest range of cover against Gram-positive and Gram-negative bacteria. Antifungal therapy with fluconazole was included for premature neonates with persistent thrombocytopenia or who cultured positive for *Candida* species.

Neonatal sepsis was divided into early onset sepsis (EOS) and late onset sepsis (LOS). Early onset sepsis, defined for preterm infants as occurring within the first 72 h of life, is often attributed to acquisition of infection during the peripartum period. Late onset sepsis, occurring after 72 h of life, is often attributed to acquisition of infection in the NICU or in the community.^10^

### Study definitions

- Contamination by commensals is a positive culture with the following organisms: corynebacterium species, micrococcus species, CONS, viridans group streptococci, propionibacterium species and bacillus species (Centers for Disease Control and Prevention definition).^11^
- Multidrug-resistant (MDR) is defined as an organism resistant to at least one agent in three or more antimicrobial categories.^18^
- Extremely drug resistant (XDR) refers to an organism resistant to at least one agent in all but two or fewer antimicrobial categories.^18^
- Carbapenem-resistant organisms are usually discussed as a special subgroup of MDR organisms.
- Repeat isolates of the same organism within 14 days were considered most likely an infection that had not been appropriately or successfully treated. In these situations, it was considered the same event. These cultures were removed from analysis in the study (‘non-significant cultures’).
- Analyses were performed using SPSS software (Version 24.0). The *p*-values less than 0.05 were considered statistically significant.

### Ethical consideration

Ethics approval was obtained from the Biomedical Research Ethics Committee of the University of KwaZulu-Natal (Ref: BE 452/16).

## Results

**FIGURE 1:**
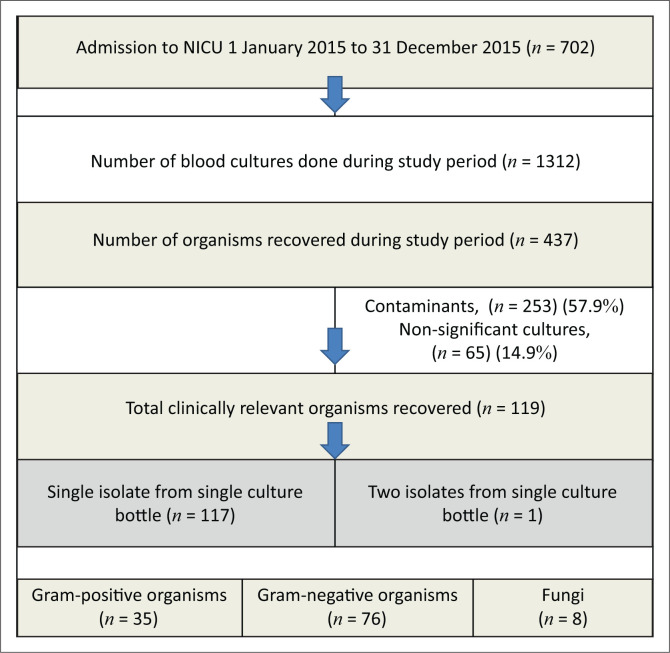
Study population.

During this 1-year study period, there were 702 admissions to the NICU (Figure 1). The microbiology laboratory received 1312 blood cultures, and a total of 437 organisms were recovered. Of the organisms recovered, 253 were considered contamination by commensals and were not included in further analyses (CONS, *n* = 176; bacillus species, *n* = 35; enterococcus species, *n* = 17; viridans group streptococci, *n* = 12; micrococcus species, *n* = 7; corynebacterium species, *n* = 1; fungal species, *n* = 5). A further 65 organisms were removed as they cultured positive within 14 days of a previous positive culture for the same organism (‘non-significant cultures’). The 119 remaining organisms were obtained from 118 samples received (one infant had two organisms grown from a single blood culture bottle). The rate of clinically relevant blood culture positivity was thus 9.0% (118/1312). The rate of clinically relevant bacteraemia amongst the patients admitted to the NICU was 16.8% (118/702).

Gram-negative bacteria were the predominantly cultured organisms making up 63.8% (76/119), with Gram-positive bacteria and fungi contributing 29.4% (35/119) and 6.7% (8/119), respectively (Table 1). Fungal infections were found significantly more in males (*p* < 0.01).

**TABLE 1:** Comparison of organisms in demographic groups.

Variable	Organism recovered						Total patients	%
	Gram-positive bacterium		Gram-negative bacterium		Fungus			
	*n*	%	*n*	%	*n*	%		
**Gender**								
Male	20	-	38	-	7	-	65	54.6
Female	12	-	28	-	1	-	41	34.4
Unknown	3	-	10	-	0	-	13	10.9

**Total**	**35**	**29.4**	**76**	**63.8**	**8**	**6.7**	**119**	**-**
**Age (in days)**								
0–3	7	-	10	-	0	-	17	14.3
4–7	7	-	19	-	1	-	27	22.7
8–28	10	-	33	-	7	-	50	42.0
28+	11	-	14	-	0	-	25	21.0
**Total**	**35**	**29.4**	**76**	**63.8**	**8**	**6.7**	**119**	**-**

Note: The sex was unknown in 13 (10.9%) patients and more positive cultures were obtained from males. Infants ranged in age from 0 to 84 days. The median age was 14 days.

Regarding Gram-positive organisms, 74.3% (26/35) were *Staphylococcus* species, 25.7% (9/35) were *Enterococcus* species and of interest, no *Streptococcus agalactiae* (Group B streptococcus [GBS]) was seen in our cohort (Table 2). Amongst the Gram-negative bacteria, 65.8% (50/76) were *Klebsiella* species and smaller percentages of patients had *Serratia* species (13.2%, 10/76), *Acinetobacter* species (7.9%, 6/76), *Enterobacter* species (3.9%, 3/76) and *Escherichia coli* (3.9%, 3/76).

**TABLE 2:** Early onset versus late onset sepsis.

Organism subtype	*n*		Total
	EOS (≤ 3 days old)	LOS (> 3 days old)	
**Gram-positive organisms (*n* = 35)**			
*Staphylococcus* species (*n* = 26):	-	-	-
*Staphylococcus capitis*	0	1	1
*Staphylococcus epidermidis*	2	8	10
*Staphylococcus aureus*	3	12	15
*Enterococcus* species (*n* = 9):	-	-	-
*Enterococcus faecium*	0	2	2
*Enterococcus faecalis*	2	5	7
**Gram-negative organisms (*n* = 76)**			
*Klebsiella* species (*n* = 50):	-	-	-
*Klebsiella oxytoca*	0	4	4
*Klebsiella pneumoniae*	7	39	46
*Serratia marcescens* (*n* = 10)	0	10	10
*Acinetobacter* species (*n* = 6):	-	-	-
*Acinetobacter lwoffii*	1	1	2
*Acinetobacter baumannii complex*	1	3	4
Enterobacter species (*n* = 3):	-	-	-
*Enterobacter aerogenes*	0	1	1
*Enterobacter cloacae*	0	2	2
*Escherichia coli* (*n* = 3)	0	3	3
*Stenotrophomonas maltophilia* (*n* = 2)	0	2	2
*Neisseria subflava* (*n* = 1)	1	0	1
*Proteus mirabilis* (*n* = 1)	0	1	1
**Fungi (*n* = 8)**			
*Candida* species:	-	-	-
*Candida parapsilosis*	0	1	1
*Candida glabrata*	0	1	1
*Candida guilliermondii*	0	1	1
*Candida albicans*	0	5	5

**Total**	**17†**	**102** ‡	**119**

† , 14.3%;

‡ , 85.7%.

EOS, early onset sepsis; LOS, late onset sepsis.

Included in Table 2 are data on Gram-positive organisms (*Staphylococcus capitis, Staphylococcus epidermidis*) that are often considered skin commensals. They were included here as they were recovered from more than one culture from the patient and were thus thought to be clinically relevant.

As noted in Table 2, most neonates had LOS (85.7%, 102/119) as compared to EOS (14.3%, 17/119). *Klebsiella pneumoniae* was the most common organism isolated in both EOS and LOS (*n* = 7 and *n* = 39, respectively). All fungi cultured were *Candida* species and were included in the LOS category.

In Table 3, *Staphylococcus* species (other than *Staphylococcus aureus*) showed poor sensitivity to all antibiotics, except for vancomycin, to which all of the Gram-positive organisms were susceptible. *Staphylococcus aureus* was highly sensitive to trimethoprim-sulphamethoxazole (94%), but minimally susceptible to clindamycin (13%). Methicillin-resistant *Staphylococcus aureus* percentage was 80% (12/15).

**TABLE 3:** Antibiotic susceptibility for all isolates.†

Variable	Total	Amik		Co-tri		Cephalo 2nd gen		Cepha 3rd gen		Cepha 4th gen		Clinda		Cipro		Clox		Colis		Erythro		Flucon		Genta		Pen		Pip – Taz		Vanco		Pen + Genta		T+A		Carba	
		*n*	%	*n*	%	*n*	%	*n*	%	*n*	%	*n*	%	*n*	%	*n*	%	*n*	%	*n*	%	*n*	%	*n*	%	*n*	%	*n*	%	*n*	%	*n*	%	*n*	%	*n*	%
**Gram-positive (*n* = 35)**																																					
*Staphylococcus* spp.	11	NT	-	4	36	NT	-	NT	-	NT	-	2	18	9	82	1	9	NT	-	1	9	NT	-	1	9	NT	-	NT	-	11	100	1	9	NT	-	NT	-
*Staphylococcus aureus*	15	NT	-	14	94	NT	-	NT	-	NT	-	2	13	14	93	3	20	NT	-	2	13	NT	-	4	27	NT	-	NT	-	15	100	4	27	NT	-	NT	-
Enterococcus	9	NT	-	0	-	NT	-	NT	-	NT	-	0	-	7	78	NT	-	NT	-	5	55	NT	-	4	45	5	55	NT	-	9	100	5	55	NT	-	NT	-
**Gram-negative (*n* = 76)**																																					
*Klebsiella*	50	50	100	16	32	8	16	10	20	41	82	NT	-	40	80	NT	-	50	100	NT	-	NT	-	17	34	0	-	39	78	NT	-	17	34	50	100	50	100
*Serratia marcescens*	10	10	100	10	100	0	-	7	70	7	70	NT	-	10	100	NT	-	0	-	NT	-	NT	-	7	70	NT	-	9	90	NT	-	7	70	10	100	10	100
*Acinetobacter*	6	3	50	1	17	1	17	2	33	2	33	NT	-	3	50	NT	-	6	100	NT	-	NT	-	3	50	0	-	1	17	NT	-	3	50	3	50	3	50
Enterobacter	3	3	100	2	66	0	-	2	66	3	100	NT	-	3	100	NT	-	3	100	NT	-	NT	-	2	66	0	-	2	66	NT	-	2	66	3	100	3	100
*Escherichia coli*	3	3	100	2	66	2	66	2	66	2	66	NT	-	2	66	NT	-	3	100	NT	-	NT	-	3	100	2	66	3	100	NT	-	3	100	3	100	3	100
*Stenotrophomonas maltophilia*	2	0	-	1	50	NT	-	1	50	1	50	NT	-	1	50	NT	-	NT	-	NT	-	NT	-	0	-	NT	-	0	-	NT	-	0	-	0	-	0	-
*Proteus mirabilis*	1	1	100	0	-	1	100	1	100	1	100	NT	-	1	100	NT	-	0	-	NT	-	NT	-	1	100	0	-	1	100	0	-	1	100	1	100	1	100
*Neisseria subflava*	1	NT	-	0	-	1	100	NT	-	NT	-	NT	-	1	100	NT	-	NT	-	NT	-	NT	-	NT	-	1	100	1	100	NT	-	1	100	NT	-	NT	-
**Fungi (*n* = 8)**																																					
*Candida* sp.	8	NT	-	NT	-	NT	-	NT	-	NT	-	NT	-	NT	-	NT	-	NT	-	NT	-	8	100	NT	-	NT	-	NT	-	NT	-	NT	-	NT	-	NT	-

† , Formula to calculate *n* (%): S/(S+R). R is number of resistant isolates and S is the number of susceptible isolates.

NT, not tested; Amik, amikacin; Co-tri, co-trimoxazole; Cephalo, cephalosporins; Clinda, clindamycin; Clox, cloxacillin; Colis, colistin; Erythro, erythromycin; Flucon, fluconazole; Genta, gentamycin; Pen, penicillin; Pip-Taz, piperacillin-tazobactam; Vanco, vancomycin.

Overall, 28.6% (10/35) of the Gram-positive organisms cultured in this unit were susceptible to the first-line antibiotic regimen. *Staphylococcus aureus* was 26.7% susceptible, *Staphylococcus* species other than S*taphylococcus aureus* were 9.1% susceptible and *Enterococcus* species were 55.6% susceptible. Thus, identification of a Gram-positive organism would generally necessitate a change in antibiotic therapy.

Amongst the Gram-negative organisms, *Klebsiella* was the most frequently isolated genus and showed fairly good sensitivity to second-line antibiotics used in the unit, namely, piperacillin-tazobactam (78%) and amikacin (100%). *Klebsiella* species were consistently susceptible (100%) to carbapenems (meropenem and imipenem) and showed high resistance to first-line empiric antibiotics (penicillin/ampicillin) (0% susceptible) and gentamycin (34% susceptible). Of the 50 *Klebsiella* species isolated, 34 (68%) were reported by the laboratory to produce an extended-spectrum beta-lactamase (ESBL).

*Acinetobacter* species showed high resistance to penicillin/ampicillin (0% susceptible), piperacillin-tazobactam (17% susceptible) and trimethoprim-sulphamethoxazole (17% susceptible). Two (33%) *Acinetobacter baumannii* were XDR, with resistance to all antibiotics except colistin. There were only three *Escherichia coli* isolated, and despite one ESBL producer, all were susceptible to first-line antibiotics. *Enterobacter* species showed high susceptibilities (> 80%) to all agents except penicillin/ampicillin (0%).

Overall, 44.7% (34/76) of the Gram-negative organisms cultured in the unit were susceptible to the first-line antibiotics, whilst most were susceptible to second-line (93.4% susceptible) and third-line (93.4% susceptible) antibiotics.

The *Candida* species isolated were all highly sensitive to fluconazole (100%).

## Discussion

During this 1-year study period, the incidence of culture-proven, clinically relevant neonatal bacteraemia was 16.8% (118/702). The rate of clinically relevant culture positivity was 8.9% (118/1312). Studies by Kayange et al. from Tanzania and Pokhrel et al. in Nepal found clinically relevant neonatal bacteraemia incidence rates higher at 39% and 20.5%, respectively.^9,19^ Local South African data, however, provided incidences of 8.5% by Motara et al. in Johannesburg and 11.5% by Morkel et al. in Cape Town.^16,20^ Variability in culture positivity exists between units. This may be attributed to differences in culture technique or administration of antibiotics prior to cultures being drawn. Gram-negative organisms were the most frequently isolated organisms in our study, similar to what has been found in many studies from developing countries.^16,19^

The timing of positive cultures also plays a role in identifying possible causative factors. Early onset sepsis is associated with organisms found in the female genital tract, often GBS and *E. coli*.^9^ Group B streptococcus was not isolated in our study. This could be attributed to intrapartum administration of antibiotics for suspected and proven premature rupture of membranes. *Escherichia coli* was also not cultured in EOS. Late onset sepsis was more common in our NICU, which differs from findings in other studies and raises concerns regarding adherence to infection prevention and control measures in our unit.^5,14^ Adherence to infection prevention and control practices can be negatively affected by rapid turnover of beds and staff shortages, as well as other unit-specific conditions. These will need to be considered, and improvements will likely require a holistic approach to establish protocols targeting improved infection-control practices.

Coagulase-negative staphylococci are usually skin commensals and are often of uncertain clinical significance. Of the 437 organisms recovered from blood cultures in the present study, 176 (40%) were CONS, which is consistent with results from other centres. Coagulase-negative staphylococci were found in 40% of positive cultures by Giannoni et al. in Switzerland and in 56% of positive blood cultures in a local South African study by Lebea et al.^21,22^ The increase in CONS could be due to poor blood culture sampling technique, but the possibility of clusters of CONS infections must also be considered. The latter may be due to the increasing numbers of very-low birth weight babies being cared for in the NICU, making septicaemia with normal skin commensal flora more commonplace.^4^ A more in-depth look at the results regarding timing of cultures and clinical correlates would have to be undertaken to try and determine factors influencing these trends.^10^ Educating members of the healthcare team on proper blood culture collection is vital in improving the quality of samples taken and ultimately the correct care of patients with positive blood cultures.^15,23^

With antibiotic resistance being a topic of global concern, knowledge of local antibiotic resistance patterns is vitally important.^7^ Our study showed that the majority of the Gram-positive organisms grown were resistant to first-line antibiotics: penicillin and gentamycin. Local data as well as data from India and Nepal showed complete resistance of *Staphylococcus* species to penicillin and gentamycin.^19,20,23^ These findings suggest that the first-line antibiotic policies may require revision to enable more sufficient coverage of the organisms present in this specific NICU.

Analysis of cultures obtained aid with guidelines for our unit. In this study, EOS was mostly caused by Gram-negative organisms, namely, *Klebsiella* species. There were no GBS. When CONS are grown, the use of the patient’s clinical picture as well as the use of septic markers should guide the use of antibiotics. A second culture positive for CONS would also suggest that the organism is not a skin commensal in that particular clinical situation. The final choice of antibiotics should be guided by the particular organism grown and its sensitivity pattern. A large majority of our patients had LOS caused by an array of Gram-negative organisms. More concerning is the number and degree of MDR organisms cultured, the large number of ESBL-producing *Klebsiella* species, as well as the presence of XDR *Acinetobacter baumannii.* A majority of these cultures form a part of the LOS category. This serves as motivation for the formation of an infection control team to make regular visits to the unit for review of positive cultures and to reinforce infection control measures in the unit.

There are several limitations to this study. As it was a laboratory audit, clinical parameters such as birth weight, presence of central lines and outcomes of patients were not known. The determination of whether certain recovered organisms (especially the frequent CONS species in our study) were clinically significant was therefore not possible. Parameters such as gestational age, presence of central lines and data on occupation rates and staff ratio per bed would have further aided the analysis and should be included in future studies. This would allow for better elucidation of the reasons for the high number of positive cultures.

A further limitation is that the data are presently 4 years old, and certainly organisms and antibiotic sensitivities may have changed in the interim. The authors do however believe it still to be relevant as practices in the mentioned NICU have changed subsequently to better address infection control. The year following the collection of these samples, a new NICU was commissioned and a Healthcare Associated Infection Quality Improvement Team was formed. The work of this team included ward rounds with the infection control nurse, microbiologist and paediatric infectious diseases specialist. The team advised the clinicians on antibiotic choice and appropriate antimicrobial interventions. The newly built NICU helped with bedspacing and improved occasional overcrowding in the unit. An audit after the initiation of these measures would certainly aid to evaluate the impact of the interventions. Furthermore, analysis of data over the course of 2–5 years may assist in identifying the spectrum of cultured organisms and antibiotic susceptibility patterns. This information may thus better guide the formulation of local empirical antibiotic regimens.

## Conclusion

Although limited by the lack of clinical data, this study does provide evidence of high numbers of a broad array of Gram-positive and Gram-negative pathogens responsible for neonatal bacteraemia. Problems with infection control in the unit are suggested by substantial levels of antimicrobial resistance, with only 39.6% (44/111) of the bacteria recovered susceptible to the first-line antibiotic regimen, as well as the predominance of LOS, a marker of nosocomial infection. Subsequent measures to improve infection control have been implemented, such as regular ward rounds with an infection control team, implementation of improved antibiotic policies and improved bedspacing in a new unit. An audit to establish the impact of these measures, whilst including important clinical information to provide context and significance, would be an important step to determine progress.
